# Transactions of the Ohio State Medical Society, Etc.

**Published:** 1851-05

**Authors:** 


TRANSACTIONS OF THE OHIO STATE MEDICAL SOCIETY, FROM ITS FIRST ORGANIZATION IN 1846, TO THE CLOSE OF ITS LAST SESSION IN 1850, WITH ADDRESSES AND ESSAYS.

We have read the proceedings of this Society with mingled feelings of pleasure and shame—pleasure in the knowledge that the profession, in Ohio, enjoys the means of progress, to be found in an annual convocation of the professional mind from all parts of the State, and shame for Kentucky that she neither has the enjoyment, nor manifests any solicitude for it.

The Transactions of the Ohio State Medical Society present several points of interest, to which we shall refer at another time. B.

